# Multiple Novel Mosquito-Borne Zoonotic Viruses Revealed in Pangolin Virome

**DOI:** 10.3389/fcimb.2022.874003

**Published:** 2022-06-29

**Authors:** Duo Zhang, Min Zheng, Ying Zhang, Guanrong Feng, Chengcheng Peng, Chenghui Li, Yiquan Li, He Zhang, Nan Li, Pengpeng Xiao

**Affiliations:** ^1^ Wenzhou Key Laboratory for Virology and Immunology, Institute of Virology, Wenzhou University, Wenzhou, China; ^2^ Guangxi Centre for Animal Disease Control and Prevention, Nanning, China; ^3^ College of Veterinary Medicine, College of Animal Science, Jilin University, Changchun, China; ^4^ College of Agriculture, Yanbian University, Yanji, China; ^5^ Academician Workstation of Jilin Province, Changchun University of Chinese Medicine, Changchun, China; ^6^ Changchun Veterinary Research Institute, Chinese Academy of Agricultural Sciences, Changchun, China

**Keywords:** metagenomic analysis, pangolin, mosquito-borne virus, viral isolation and identification, phylogenetic analysis

## Abstract

Swab samples were collected from 34 pangolins in Guangxi Province, China. Metavirome sequencing and bioinformatics approaches were undertaken to determine the abundant viral sequences in the viromes. The results showed that the viral sequences belong to 24 virus taxonomic families. To verify the results, PCR combined with phylogenetic analysis was conducted. Some viral sequences including Japanese encephalitis virus (JEV), Getah virus (GETV), and chikungunya virus (CHIKV) were detected. On the basis of the metavirome analysis, seven segments belonging to JEV were further identified through PCR amplification. Sequence comparison showed that, among seven sequences, JEV-China/P2020E-1 displayed the highest nucleotide (80.6%), with the JEV isolated in South Korea, 1988, and all of which belonging to genotype III. Seven CHIKV sequences were detected, with the highest homology (80.6%) to the *Aedes africanus* in Côte d’Ivoire, 1993. Moreover, passage from BHK-21 to Vero cells makes the newly isolated CHIKV-China/P2020-1 more contagious. In addition, the newly verified GETV sequences shared 86.4% identity with the 1955 GETV isolated from Malaysia. Some sudden and recurrent viruses have also been observed from the virome of pangolin in Guangxi Province, China; hence, dissemination tests will be implemented in the future.

## 1 Introduction

Pangolins are a newly discovered intermediate vector of several pathogenic viruses, including Sendai virus, coronavirus, and severe acute respiratory syndrome coronavirus 2 (SARS-CoV-2), among others, which have become a huge threat to human life and health (Ping et al., 2019; Supaporn et al., 2021). Interestingly, viruses such as the pangolin-CoVs have been found in Guangxi Province, China (Chase et al., 2020; Min-Sheng et al., 2021). Therefore, regional viral monitoring in pangolins is an urgent need. Generally, known viruses are detected using multiple PCR methods (Morales et al., 2017; Ibrahim et al., 2021; La et al., 2021). However, compared with the traditional methods, Illumina sequencing is a more time-efficient and labor-saving technique for the detection of low viral loads (He et al., 2013; Cabral-Castro et al., 2016; Martínez-Puchol et al., 2020). Therefore, Illumina sequencing may be beneficial for examining the highly contagious and pathogenic viruses in viral hosts and for avoiding the omission of unknown viruses.

The present study aimed to establish an effective approach for monitoring pangolin-borne viruses in Guangxi Province, China, offering valuable information on virus isolation, identification, and surveillance. We firstly obtained the diversity and abundant viromes from pangolins in Guangxi Province through Illumina sequencing combined with PCR. In addition, we further confirmed the discovery of Japanese encephalitis virus (JEV) and the new Getah virus (GETV) and isolated chikungunya virus (CHIKV). Our preliminary exploration of the pangolin-borne virome could provide technical support in the discovery of viruses and in the diversity of viruses from pangolins in China and other countries.

## 2 Materials and Methods

### 2.1 Pangolin Sample Collection

Swab samples were collected from 34 healthy pangolins in Guangxi Province, China. The pangolins were raised in cages and fed with the same diet in the same feeding environment. The obtained anal and throat swab samples were collected from pangolins of three genetic generations—generation I (GI), generation II (GII), and generation III (GIII)—which were offspring from different parents. The pangolin samples were grouped by generation (**Table 1**) and preserved at −80°C. Our study was approved by the Research Ethics Committee of Wenzhou University. Experiments related to viral operation were conducted in a P-3 biosafety laboratory.

**Table 1:** Pangolin samples used in the metagenomic analysis and data from Illumina sequencing.

Sample	Species	Number	Location	Total read number	Average (nt)	Viral reads	Viral contigs
GI	Swab^a^	10	Nanning City^b^	6,147,523	177.39	1,063,227	5,241
GII	Swab^a^	12	Nanning City^b^	7,026,342	186.15	862,396	6,047
GIII	Swab^a^	12	Nanning City^b^	7,364,514	182.47	1,137,549	4,634
Total/average		34		20,538,379	182.21	3,063,172	15,922

**Figure 1:**
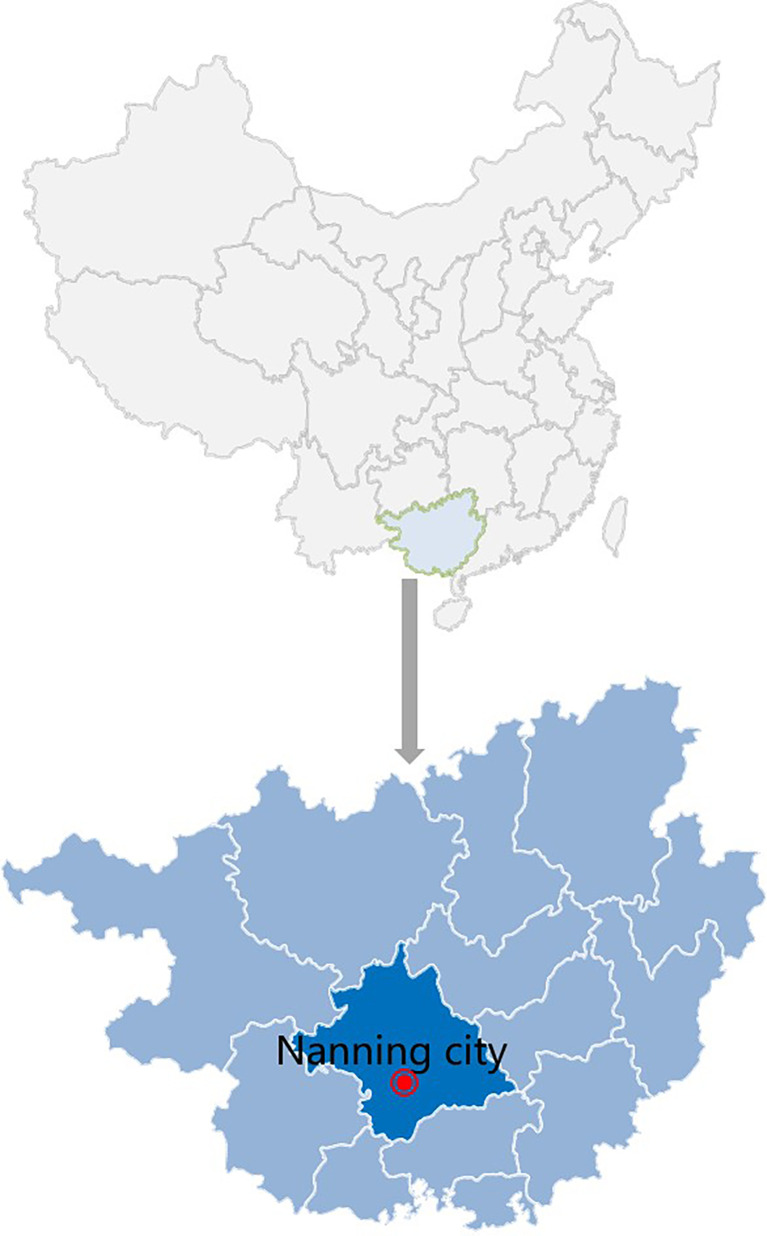
Sample collection site in Guangxi Province, China (2020). The sample collection site was labeled with a solid red circle.

GI, GII, and GIII denote generations I, II, and II, respectively.^a^Swabs included anal and throat swabs.^b^The collection site (**Figure 1**) was Nanning City (29°32¢ N, 103°70¢ E), Guangxi Province

### 2.2 Sample Preparation and Metaviral Sequencing

All experiments employed in metavirome sample preparation and in metaviral sequencing were referenced to the approaches in our previous research (Pengpeng et al., 2018; Guanrong et al., 2022). The barcode DNA used in metavirome analysis is displayed in **Table 2**.

**Table 2:** Barcode DNA used in the metagenomic analysis (He et al., 2013).

Primer type	Primer number	Primers (5′–3′)
Anchored Random primers	RT1	GCCGGAGCTCTGCAGATATCNNNNNN
	RT2	GTATCGCTGGACACTGGACCNNNNNN
	RT3	ATCGTCGTCGTAGGCTGCTCNNNNNN
Barcode primers	Primer1	GCCGGAGCTCTGCAGATATC
	Primer2	GTATCGCTGGACACTGGACC
	Primer3	ATCGTCGTCGTAGGCTGCTC

### 2.3 Sequence Alignment

We aligned the sequences from Illumina sequencing with non-redundant databases and virus reference databases through BLASTx and BLASTn in GenBank (https://www.ncbi.nlm.nih.gov/genbank/). Statistical significance was reached when *E* ≤ 10e^−5^ of BLAST hits. Sequences belonging to bacteria and eukaryotes were removed and virus-like sequences were analyzed.

### 2.4 Virus-Like Sequence Identification

The viral sequences with known sequences listed in the GenBank database were compared. Subsequently, specific primers were designed and synthesized according to the matching positions (**Table 3**) to detect virus-like sequences. Abstraction of viral nucleic acid was conducted through a viral nucleic acid extraction kit (BIOER, Hangzhou, China), and amplification of viral nucleic acid was performed using PCR Master Mix (Trans, Beijing, China) and specific primers.

**Table 3:** Primer pairs used in PCR identification.

Primer name	Primers (5′–3′)	Product (bp)
JEV-China/P2020E-1/2/3-F	TTTAATTGTCTGGGAATGGGCA	1,500
JEV-China/P2020E-1/2/3-R	AGCATGCACATTGGTCGCTAAGA	
JEV-China/P2020NS4a-1/2/3/4-F	TCAGCCATTAGCTTCATAGAG	378
JEV-China/P2020NS4a-1/2/3/4-R	CCTCTGTTTTTCCGGTTCTGG	
CHIKV-China/P2020E1-1/2/3/4-F	TACGATCAGGTAACTGTGAACC	1,317
CHIKV-China/P2020E1-1/2/3/4-R	GTGCCTGCTAAACGACACGCATAG	
CHIKV-China/P2020NS1-1/2/3-F	ATGGATCCTGTGTACGTGCAC	1,605
CHIKV-China/P2020NS1-1/2/3-R	TGCGCCCGCTCTGTCCTCAAG	
GETV-China/P2020NS3-1/2-F	GCACCGTCATACAGCGTCCGC	1,572
GETV-China/P2020NS3-1/2-R	CGCGCCAGCGCTGCCTAGTGA	
CHIKV-F^a^	TACGATCAGGTAACTGTGAACC	1,317
CHIKV-R^a^	GTGCCTGCTAAACGACACGCATAG	

JEV, Japanese encephalitis virus; CHIKV, chikungunya virus; GETV, Getah virus.^a^Primers used in CHIKV identification after virus isolation.

### 2.5 Phylogenetic Analysis

After amplification, the PCR products were sequenced, which were aligned with the representative viral sequences through ClustalW 2.0. Thereafter, phylogenetic trees using a maximum-likelihood algorithm with 1,000 bootstrap replicates were built with MEGA software, version 7.0.

### 2.6 Virus Isolation

BHK-21 and Vero cells were used in this study. The cell lines were cultured using Dulbecco’s modified Eagle’s medium (DMEM; HyClone, Logan, UT, USA), in which 8% fetal bovine serum (FBS; Gibco, Grand Island, NY, USA) and 1.2% penicillin and streptomycin (Pen Strep.; Gibco, Grand Island, NY, USA) were supplemented. Cells were cultivated at 37°C, in 5% CO_2_ conditions in the thermostat. PCR-positive pangolin samples were selected for virus isolation. Briefly, the grinding liquid supernatant of the pangolin samples was mixed 1:2 with DMEM, which was supplemented with 2% FBS. In addition, the liquid mixture was inoculated on monolayer BHK-21 cells with a growth density of 70%–80%, followed by culture and observation for 5–7 days. The cytopathic effect (CPE) caused by viral infection was monitored daily. Cultures were blind passaged three times when no CPE was observed.

### 2.7 Verification of the Isolated Virus

A PCR assay was performed for identification of the isolated CHIKV-China/P2020-1. According to the specific sequence of the CHIKV envelope 1 (*E1*) gene, the primers were synthesized as designed (**Table 3**). Western blot analysis further verified the reliability with the primary antibody of the anti-E1 monoclonal antibody (Abcam, Cambridge, UK) and the second antibody of the horseradish peroxidase (HRP)-conjugated rabbit anti-mouse antibody (Trans, Beijing, China).

### 2.8 Negative Staining Electron Microscopy Observation

The newly isolated CHIKV was inoculated on BHK-21 cells and induced CPE. The cells were then harvested, subjected to three freeze–thaw cycles, and subsequently centrifuged at 10,000 × *g* for 12 min. Viral suspension was then obtained with centrifugation at 60,000 × *g* for 4 h. After gently removing the supernatant, the virus precipitate was obtained. A 6% glutaraldehyde fixative (pH 7.2) was mixed with an equal amount of DMEM, and then the precipitate was resuspended using the liquid mixture, from which 30 μl was drawn and added to a copper mesh. Finally, for negative staining, one drop of 3% phosphotungstic acid was added. Observation was performed using an electron microscope.

### 2.9 Viral Titer Detection

CHIKV was cultured and passaged in BHK-21 cells. The viral titers were tested at the 5th and the 10th generation. The viral fluid was serially diluted 10 times, followed by inoculation on monolayer BHK-21 cells with a growth density of 70%–80%, with incubation at 37°C for 120 h. Each dilution was established with eight holes and the CPE of CHIKV was observed. The TCID_50_ (50% tissue culture infective dose) was determined according to the Reed–Muench approach (Krah et al., 1991).

### 2.10 Viral Variation Analysis

BHK-21 cells produced CPE caused by the newly isolated CHIKV in the 5th and 10th generations. The total RNA of the virus was isolated from the lysate with an RNA extraction kit following the manufacturer’s instructions (Bioer Technology, Hangzhou, China). When passaged in Vero from BHK-21 cells of CHIKV (5th and 10th), which viral RNA was employed as amplification template with SuperScript™ III (Invitrogen) in accordance with the directions for use. The *E1* sequences of the 5th and 10th generations of CHIKV were amplified, sequenced, and then aligned using MEGA 7.0.

### 2.11 Statistical Analysis

SPSS 17.0 was applied for statistical analysis, using *t*-test for comparisons between groups. Each experiment was repeated three times. *P* < 0.05 was considered a significant difference. Metagenomic sequencing data have been registered in GenBank (accession nos. SRR15264668, SRR15264669, and SRR15264670). The amplified sequences were also accepted by GenBank, with accession numbers: JEV-China/P2020E-1/2/3(OM416153–OM416151), JEV-China/P2020NS4a-1/2/3/4 (OM416150–OM416147), CHIKV-China/P2020E1-12/3/4 (OM416163–OM416160), CHIKV-China/P2020NS1-1/2/3 (OM416159–OM416157), and GETV-China/P2020NS3-1/2/3 (OM416156–OM416154).

## 3 Results

### 3.1 Pangolin Virome

**Figure 2:**
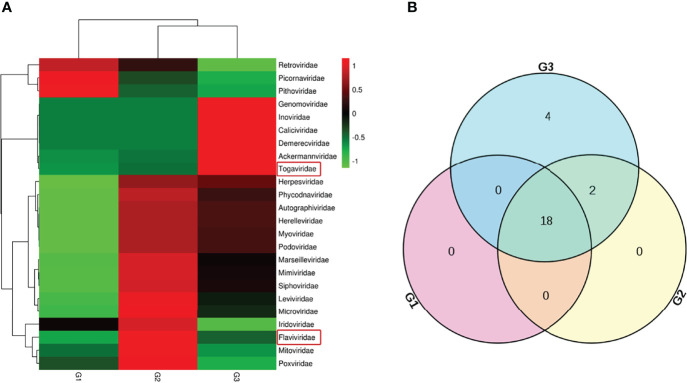
Abundance and relationship of the virus families in the three samples. **(A)** Classification of the viral sequences was according to the virus family, with the relative abundance shown in a heat map. **(B)** Number of virus families in the three samples laid out in a Venn diagram.

Host sequences along with the barcode DNA were excluded to acquire clean data from pangolin viromes. Using Illumina sequencing, 20,538,379 reads were obtained, with an average read length of 182.21 nt (**Table 1**). The viral sequences from GI, GII, and GIII occupied 17.30%, 12.27%, and 15.45%, respectively. The relative abundance of the virus families is displayed in **Figure 2A**, with the intersection of three samples shown in **Figure 2B**. There were 18 virus families observed in GI, 20 in GII, and 24 in GIII. Interestingly, representative viral sequences from two families—Flaviviridae and Togaviridae—were obtained in all three groups.

### 3.2 PCR Verification of the Pangolin Virome

After contig assembly using SOAPdenovo, 15,922 viral contigs were generated. Some virus-like contigs showed high sequence similarity; for instance, 103 JEV-like contigs had a read coverage of 42× (275–861 nt), 136 CHIKV-like contigs had a read coverage of 39× (236–917 nt), and 67 GETV-like contigs had a read coverage of 23× (219–875 nt).

The JEV-like contigs shared 80.7%–97.3% similarity with the nucleotides of the known JEV sequences, the CHIKV-like contigs had 85.6%–92.8% similarity with the nucleotides of the known CHIKV sequences, while the GETV-like contigs had 84.1%–95.2% similarity with the nucleotides of the known GETV sequences. To further verify the results of the metavirome sequencing, the identified viruses were amplified by specific primers. The verification results are detailed in the following sections.

#### 3.2.1 PCR Verification of JEV

**Figure 3:**
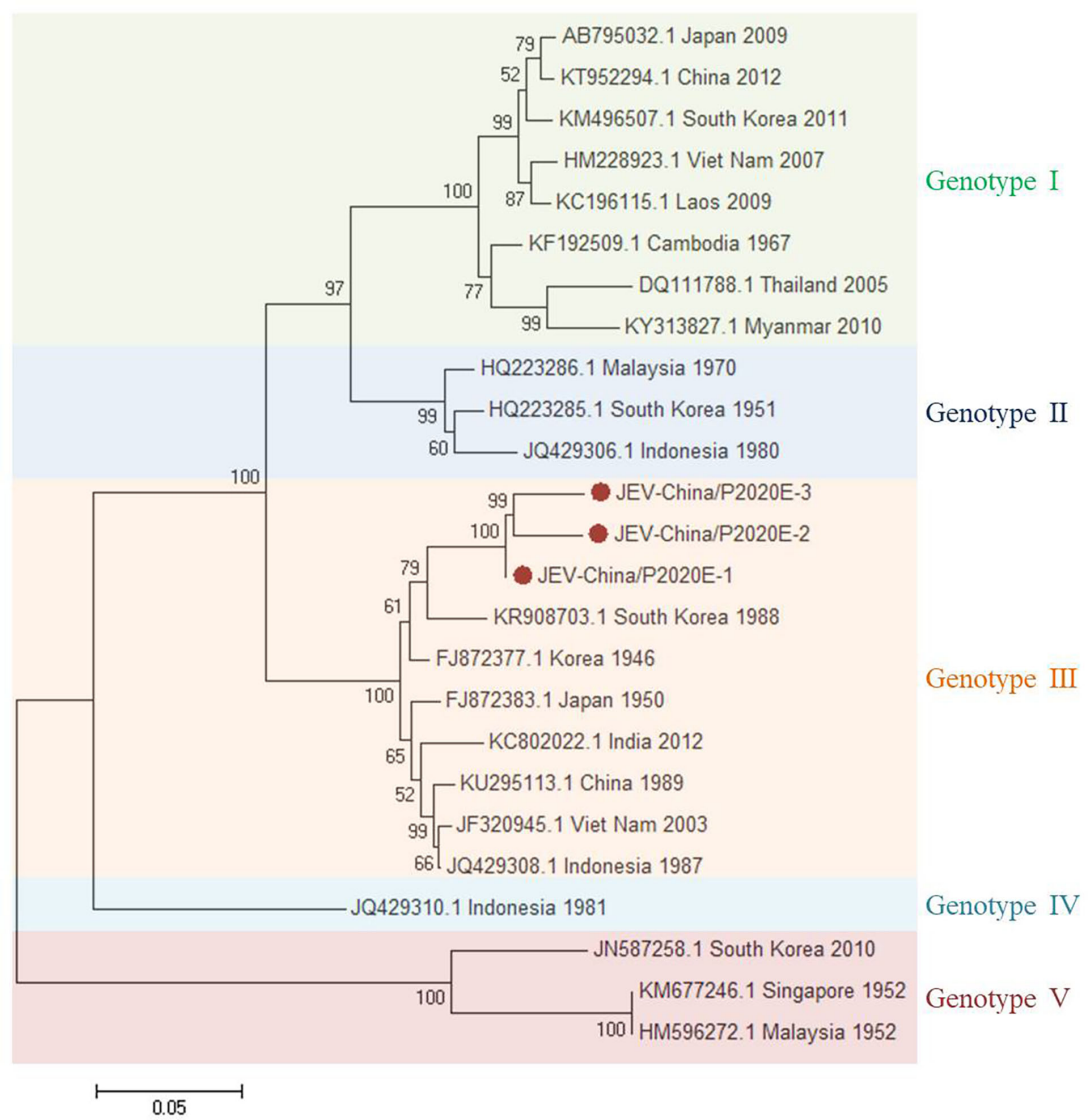
Phylogenetic trees based on JEV-E. The trees were constructed according to the alignments of the nucleotide sequences of the *E* gene of Japanese encephalitis virus (JEV). The maximum likelihood method was applied using MEGA 7.0, in which the bootstrap value was set to 1,000. *Solid red circles* denote the viral genes detected in this study.

The viral amplicons in the samples from GII were highly homologous to JEV assigned to *Flavivirus* of Flaviviridae. Three 1,500-nt-long segments (JEV-China/P2020E-1/2/3) and four 378-nt-long segments (JEV-China/P2020NS4a-1/2/3/4) were amplified, which shared ~95.5%–97.5% and ~92.1%–96.0% nucleotide identity and ~90.0%–94.0% and ~84.1%–93.7% amino acid (aa) with each other, respectively. The viral segments were compared with the sequences published in GenBank using BLASTN. The results showed that the segments had high homology with the *E* sequences and the non-structural protein 4a (NS4a) of JEV from South Korea discovered in 1988; the identities of the nucleotides were at least 80.6% and 82.7% and those of aa were 74.7% and 82.4%, respectively. These results indicate that these viral genes came from the same JEV variant (**Figure 3**). Phylogenetic analysis demonstrated that the verified JEV segments belong to genotype III.

#### 3.2.2 PCR Verification of CHIKV

**Figure 4:**
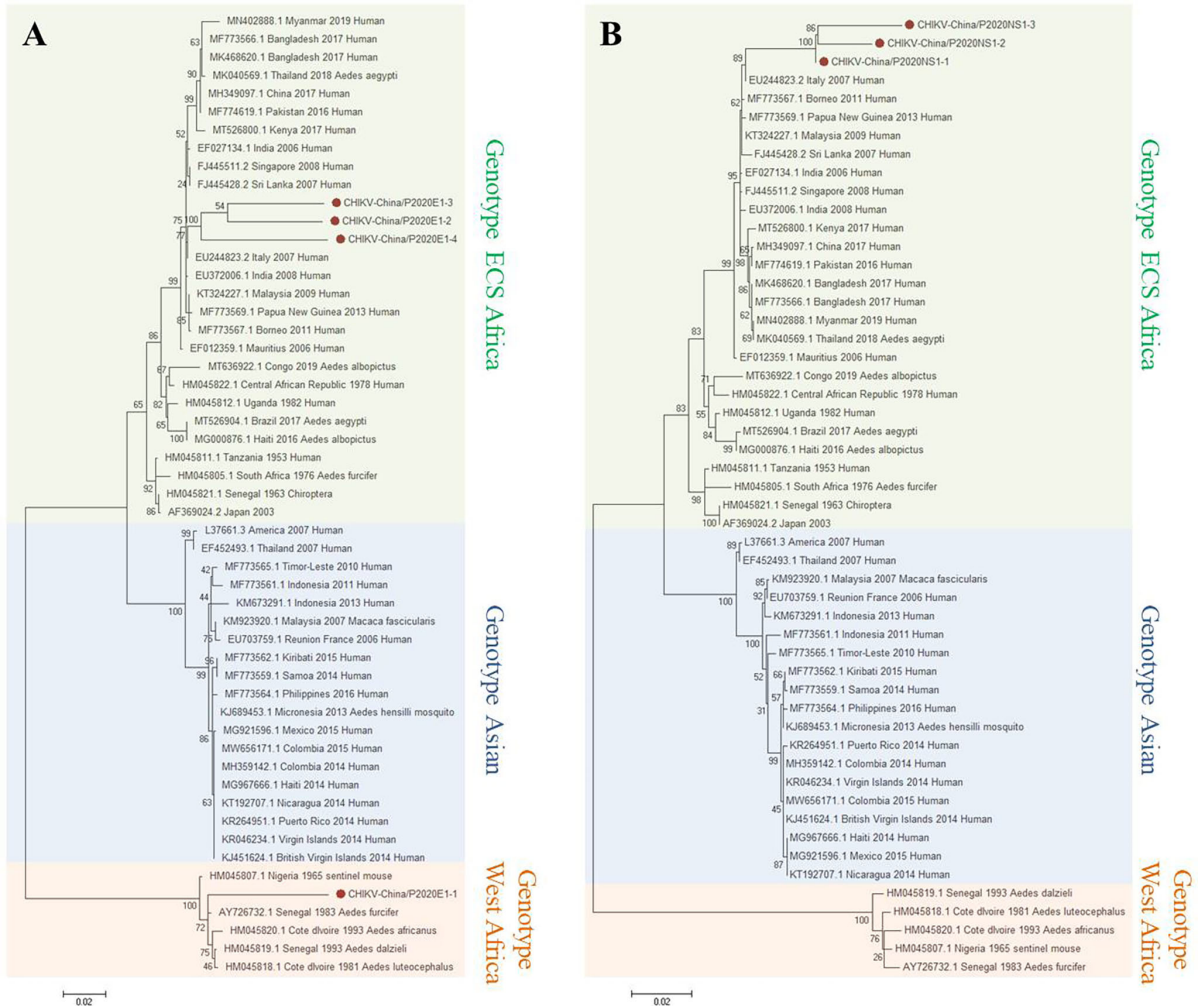
Phylogenetic trees based on CHIKV-E1/NS1. The trees were constructed according to the alignments of the nucleotide sequences of the *E1* gene **(A)** and the NS1 gene **(B)** of chikungunya virus (CHIKV). The maximum likelihood method was applied using MEGA 7.0, in which the bootstrap value was set to 1,000. *Solid red circles* denote the viral genes detected in this study.

Based on the results of the viral sequence alignment, the viral amplicons in the samples from GIII were highly homologous to CHIKV belonging to *Alphavirus* of Togaviridae. Four fragments of 1,317 nt in length (CHIKV-China/P2020E1-1/2/3/4) and three fragments of 1,605 nt in length (CHIKV-China/P2020NS1-1/2/3) were amplified and shared ~77.7%–91.6% and ~95.6%–98.2% nucleotide and ~74.9%–82.4% and ~90.5%–95.9% aa identities with each other. In comparison with the sequences in GenBank, the segments had high homology with the *E1* sequences and the non-structural protein 1 (NS1) of CHIKV from Cote d’Ivoire discovered in 1993. The identities of the nucleotides were at least 80.6% and 82.7% and those of aa were 83.6% and 85.4%, respectively. It indicated that these viral genes came from the same CHIKV variant (**Figure 4**). Phylogenetic analysis indicated that the identified CHIKV sequences belong to genotype ECS and West Africa viral genes.

#### 3.2.3 PCR Verification of GETV

**Figure 5:**
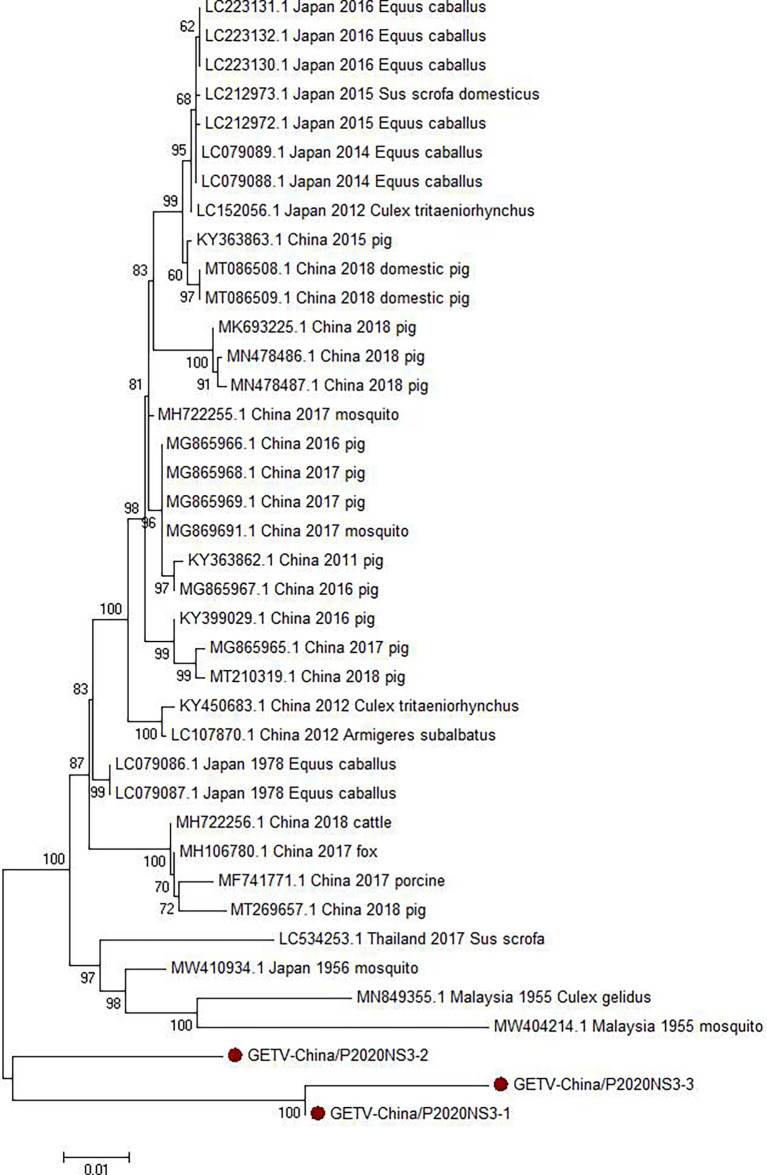
Phylogenetic trees based on GETV-NS3. The trees were constructed according to the alignments of the nucleotide sequences of the NS3 protein of Ross River virus (RRV). The maximum likelihood method was applied using MEGA 7.0, in which the bootstrap value was set to 1,000. *Solid red circles* denote the viral genes detected in this study.

Viral amplicons in the samples from GIII were highly homologous to GETV, which belong to *Alphavirus* of Togaviridae, in good agreement with the results of the contig alignment. Three 1,572-nt-long segments (GETV-China/P2020NS3-1/2/3) were amplified and shared ~90.3%–97.3% nucleotide and ~78.4%–93.3% aa identities with each other. In comparison with the sequences in GenBank, the segments had high homology with the non-structural protein 3 (NS3) sequence of GETV from human discovered in Malaysia in 1955. The identity of the nucleotides was at least 86.4% and that of aa was 81.3% (**Figure 5**).

### 3.3 Isolation and Identification of CHIKV

**Figure 6:**
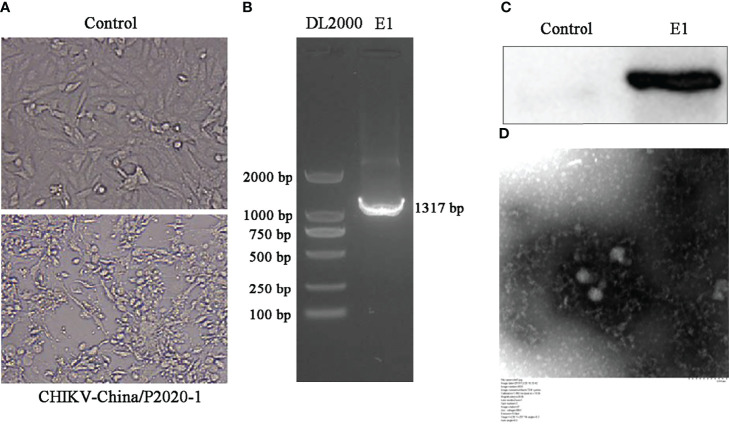
Identification of CHIKV-China/P2020-1 isolated in Nanning City, Guangxi Province. **(A)** The cytopathic effect (CPE) of CHIKV-China/P2020-1 was observed after infection in BHK-21 cells. **(B)** Identification of CHIKV-China/P2020-1 by PCR. **(C)** Identification of CHIKV-China/P2020-1 by Western blot. **(D)** Negative-stain electron microscopy of CHIKV-China/P2020-1 particles.

After the amplification of CHIKV in sample GIII, we examined all anal swab samples from GIII in order to isolate CHIKV. Firstly, the isolated virus was tested using PCR, and Western blot was conducted to determine the viral expression level (E1 protein). As shown in **Figure 6**, the *E1* sequence and the protein were both positively expressed in viral-infected cells, but not in mock-infected cells. CHIKV-like particles can be observed through negative-stain electron microscopy, displaying diameter of approximately 70 nm, with tiny protrusions on the surface, which further confirmed the above analysis.

### 3.4 Viral Titer of CHIKV-China/P2020-1

CHIKV-China/P2020-1 was passaged on BHK-21 cells and then the viral titer tested. Viral titers of 4.17 × 10^4^ and 2.65 × 10^4^ TCID_50_/0.1 ml were detected in the 5th and 10th generations, respectively, indicating that multiple passages might induce viral titer changes.

### 3.5 Variability of CHIKV-China/P2020-1

The CHIKV-China/P2020-1 *E1* genes of the 5th and 10th generations were sequenced and aligned. Sequence analysis revealed that the *E1* sequences of the 5th generation shared 99.2% homology with the 10th generation in nucleotide sequence. CHIKV-China/P2020-1 was subsequently used to infect Vero cells, with the *E1* genes of the 5th and 10th generations sequenced and their homology analyzed. Nucleotide comparison analysis revealed that 8 nucleotide sites in the *E1* genes of the 5th generation and 17 sites in the 10th generation showed polymorphisms. Comparison of the amino acid sequences exhibited *E1* gene mutations identified in both 5th and 10th generations: mutation from A (alanine) to V (valine) at 226 aa, which displayed the same type and site. In addition, the viral titers in the 5th and 10th generations in Vero cells were 1.08 × 10^5^ and 2.63 × 10^5^ TCID_50_/0.1 ml, respectively, which were markedly higher than those in BHK-21 cells.

## 4 Discussion

Pangolins have been confirmed to be intermediate hosts of SARS-CoV-2 (Tommy et al., 2020; Kangpeng et al., 2020; Lamia et al., 2020). However, many potential viruses carried by pangolins have not yet been discovered. Illumina sequencing combined with bioinformatics analysis is more likely to be effective than traditional methods (Zhang et al., 2018; Marzoli et al., 2021; Thannesberger et al., 2021). Moreover, a lot of viruses derived from numerous species have been detected using this approach (Sadeghi et al., 2018; Santos et al., 2021). Therefore, the composition of the pangolin virome can be revealed using Illumina sequencing. In this study, we made use of Illumina sequencing and detected as many as 24 virus families in pangolin samples. However, the percentages of the viral sequences obtained from the three groups differed widely, which may be linked to vertical transmission. Nevertheless, our findings support the discovery of the existence and evolution of pangolin viromes in Guangxi Province, China.

RT-PCR and PCR were performed to validate the reliability of the results of Illumina sequencing. The results showed that JEV and GETV were amplified in pangolin samples, which was the first time that these viral sequences were discovered in pangolins from Guangxi Province, China, suggesting that an epidemic of JEV and GETV might have existed here. Furthermore, the unidentified virus detected during Illumina sequencing might be limited by the sample/collection site shortage.

The phenomenon of CHIKV and GETV being amplified simultaneously in pangolin samples revealed the co-transmission and probably co-infection of the two viruses in Guangxi Province. While our current data were insufficient for proving the co-infection of the viruses, the discovery of the co-circulation of GETV and CHIKV is still of great significance, which put forward a new demand for the prevention and control of CHIKV.

Based on the isolation and identification of CHIKV, it was considered that the virus was still propagating and circulating in Guangxi Province. Further sequence alignment results showed that *E1* gene mutations in CHIKV-China/P2020-1 were identified in both 5th and 10th generations: mutation from A (alanine) to V (valine) at 226 aa, which had the same type and site. Research indicated that the E1-A226V mutation at position 226 in the E1 protein can considerably enhance the adaptability of CHIKV to *Aedes albopictus* (Agbodzi et al., 2021). Our subsequent study demonstrated that the viral titers of the 5th and 10th generations in Vero cells were markedly higher than those in BHK-21 cells, revealing that interspecies transmission can make CHIKV more contagious, thus increasing the threat to human health. The enhanced cross-host infectivity of CHIKV, combined with the discovery of the mosquito-borne CHIKV in pangolins, suggest that the spread of CHIKV will bring about major challenges to virus prevention and control.

The detection of viruses in pangolins from Guangxi Province in this study further revealed the possibility that they carry human-infecting viruses. However, the present research only explored a fraction of the pangolin viromes in this region, which needs to be extended in more countries or regions in order to explore the abundance and diversity of the pangolin viromes. Overall, our study has provided valuable insights into virus isolation and the identification of CHIKV and a new GETV, which may be meaningful to viral epidemiology study.

## Data Availability Statement

The datasets presented in this study can be found in online repositories. The names of the repository/repositories and accession number(s) can be found in the article/Supplementary Material.

## Ethics Statement

Ethical review and approval was not required for the study of pangolin participants, in accordance with the local legislation and institutional requirements.

## Author Contributions

PX and NL conceived and designed the experiments. DZ, GF, CP, CL, and HZ performed the experiments. PX, YZ, and YL analyzed the data. MZ contributed reagents/materials/analysis tools. PX and NL wrote the paper. All authors contributed to the article and approved the submitted version.

## Funding

This work was mainly supported by the National Natural Science Foundation of China (grant no. 32002312) and partly supported by the Natural Science Foundation of Zhejiang Province (grant no. LQ21H160001) and the Science and Technology Plan Project of Wenzhou, Zhejiang, China (grant nos. Y20210080 and Y2020103).

## Conflict of Interest

The authors declare that the research was conducted in the absence of any commercial or financial relationships that could be construed as a potential conflict of interest.

## Publisher’s Note

All claims expressed in this article are solely those of the authors and do not necessarily represent those of their affiliated organizations, or those of the publisher, the editors and the reviewers. Any product that may be evaluated in this article, or claim that may be made by its manufacturer, is not guaranteed or endorsed by the publisher.

## References

[B1] Agbodzi B. Yousseu F. Simo F. Kumordjie S. Yeboah C. Mosore M. T.. (2021). Chikungunya Viruses Containing the A226V Mutation Detected Retrospectively in Cameroon Form a New Geographical Subclade. Int. J. Infect. Dis. 113, 65–73. doi: 10.1016/j.ijid.2021.09.058 34592442

[B2] Cabral-Castro M. J. Peralta R. Cavalcanti M. G. Puccioni-Sohler M. Carvalho V. L. da Costa Vasconcelos P. F.. (2016). A Luminex-Based Single DNA Fragment Amplification Assay as a Practical Tool for Detecting and Serotyping Dengue Virus. J. Virol. Methods 236, 18–24. doi: 10.1016/j.jviromet.2016.07.003 27393681

[B3] Chase W. N. Zachary A. Tony L. G. Chen M. Chen-Hao K. Christina L.. (2020). Dynamically Evolving Novel Overlapping Gene as a Factor in the SARS-CoV-2 Pandemic. Elife. 9, e59633. doi: 10.7554/eLife.59633 33001029 PMC7655111

[B4] Guanrong F. Jinyong Z. Ying Z. Chenghui L. Duo Z. Yiquan L.. (2022). Metagenomic Analysis of Togaviridae in Mosquito Viromes Isolated From Yunnan Province in China Reveals Genes From Chikungunya and Ross River Viruses. Front. Cell. Infect. Microbiol. 12. doi: 10.3389/fcimb.2022.849662 PMC8878809 35223559

[B5] He B. Li Z. Yang F. Zheng J. Feng Y. Guo H.. (2013). Virome Profiling of Bats From Myanmar by Metagenomic Analysis of Tissue Samples Reveals More Novel Mammalian Viruses. PloS. One 8, e61950. doi: 10.1371/journal.pone.0061950 23630620 PMC3632529

[B6] Ibrahim I. Iliya S. N. Maryam A. Gloria C. Andreas D. Ayo Y. S. (2021). Genetic Diversity of Dengue Virus Serotypes Circulating Among Aedes Mosquitoes in Selected Regions of Northeastern Nigeria. One Health 13, 100348. doi: 10.1016/j.onehlt.2021.100348 34825044 PMC8605110

[B7] Kangpeng X. Junqiong Z. Yaoyu F. Niu Z. Xu Z. Jie-Jian Z.. (2020). Isolation of SARS-CoV-2-Related Coronavirus From Malayan Pangolins. Nature. 583 (7815), 286–289. doi: 10.1038/s41586-020-2313-x 32380510

[B8] Krah D. L. (1991). A Simplified Multiwell Plate Assay for the Measurement of Hepatitis A Virus Infectivity. Biologicals. 19, 223–227. doi: 10.1016/1045-1056(91)90039-M 1659431

[B9] La R. G. Mancini P. Bonanno F. G. Veneri C. Iaconelli M. Lucentini L.. (2021). Rapid Screening for SARS-CoV-2 Variants of Concern in Clinical and Environmental Samples Using Nested RT-PCR Assays Targeting Key Mutations of the Spike Protein. Water. Res. 197, 117104. doi: 10.1016/j.watres.2021.117104 33857895 PMC8018700

[B10] Lamia W. Nimit J. Andrew Z. F. Massa J. S. Karen L. A. Matthew J. M.. (2020). An Extensive Meta-Metagenomic Search Identifies SARS-CoV-2-Homologous Sequences in Pangolin Lung Viromes. mSphere. 5 (3), e00160–e00120. doi: 10.1128/mSphere.00160-20 32376697 PMC7203451

[B11] Martínez-Puchol S. Rusiñol M. Fernández-Cassi X. Timoneda N. Itarte M. Andrés C.. (2020). Characterisation of the Sewage Virome: Comparison of NGS Tools and Occurrence of Significant Pathogens. Sci. Total. Environ. 713, 136604. doi: 10.1016/j.scitotenv.2020.136604 31955099

[B12] Marzoli F. Forzan M. Bortolotti L. Pacini M. I. Rodríguez-Flores M. S. Felicioli A.. (2021). Next Generation Sequencing Study on RNA Viruses of Vespa Velutina and Apis Mellifera Sharing the Same Foraging Area. Transbound Emerg. Dis. 68 (4), 2261–2273. doi: 10.1111/tbed.13878 33063956

[B13] Min-Sheng P. Jian-Bo L. Zheng-Fei C. Hang L. Xiaolu T. Ruochen Y.. (2021). The High Diversity of SARS-CoV-2-Related Coronaviruses in Pangolins Alerts Potential Ecological Risks. Zool. Res. 42 (6), 834–844. doi: 10.24272/j.issn.2095-8137.2021.334 34766482 PMC8645874

[B14] Morales M. A. Fabbri C. M. Zunino G. E. Kowalewski M. M. Luppo V. C. Enría D. A.. (2017). Detection of the Mosquito-Borne Flaviviruses, West Nile, Dengue, Saint Louis Encephalitis, Ilheus, Bussuquara, and Yellow Fever in Free-Ranging Black Howlers (Alouatta Caraya) of Northeastern Argentina. PLoS. Negl. Trop. Dis. 11 (2), e0005351. doi: 10.1371/journal.pntd.0005351 28187130 PMC5330535

[B15] Pengpeng X. Jicheng H. Ying Z. Chenghui L. Xiaofang G. Shubo W.. (2018). Metagenomic Analysis of *Flaviviridae* in Mosquito Viromes Isolated From Guangxi Province in China Reveals Genes From Dengue and Zika Viruses. Front. Cell. Infect. Microbiol. 8. doi: 10.3389/fcimb.2018.00359 PMC6207848 30406038

[B16] Ping L. Wu C. Jin-Ping C. (2019). Viral Metagenomics Revealed Sendai Virus and Coronavirus Infection of Malayan Pangolins (*Manis Javanica*). Viruses. 11 (11), 979. doi: 10.3390/v11110979 PMC6893680 31652964

[B17] Sadeghi M. Altan E. Deng X. Barker C. M. Fang Y. Coffey L. L.. (2018). Virome of > 12 Thousand Culex Mosquitoes From Throughout California. Virology. 523, 74–88. doi: 10.1016/j.virol.2018.07.029 30098450

[B18] Santos P. D. Ziegler U. Szillat K. P. Szentiks C. A. Strobel B. Skuballa J.. (2021). In Action-an Early Warning System for the Detection of Unexpected or Novel Pathogens. Virus. Evol. 7 (2), veab085. doi: 10.1093/ve/veab085 34703624 PMC8542707

[B19] Supaporn W. Chee W. T. Patarapol M. Prateep D. Feng Z. Yutthana J.. (2021). Evidence for SARS-CoV-2 Related Coronaviruses Circulating in Bats and Pangolins in Southeast Asia. Nat. Commun. 12 (1), 972. doi: 10.1038/s41467-021-21240-1 33563978 PMC7873279

[B20] Thannesberger J. Rascovan N. Eisenmann A. Klymiuk I. Zittra C. Fuehrer H. P.. (2021). Viral Metagenomics Reveals the Presence of Novel Zika Virus Variants in *Aedes* Mosquitoes From Barbados. Parasitol. Vectors. 14 (1), 343. doi: 10.1186/s13071-021-04840-0 PMC8244189 34187544

[B21] Tommy T. L. Na J. Ya-Wei Z. Marcus H. S. Jia-Fu J. Hua-Chen Z.. (2020). Identifying SARS-CoV-2-Related Coronaviruses in Malayan Pangolins. Nature. 583 (7815), 282–285. doi: 10.1038/s41586-020-2169-0 32218527

[B22] Zhang D. Lou X. Yan H. Pan J. Mao H. Tang H.. (2018). Metagenomic Analysis of Viral Nucleic Acid Extraction Methods in Respiratory Clinical Samples. BMC. Genomics 19 (1), 773. doi: 10.1186/s12864-018-5152-5 30359242 PMC6202819

